# Effects of high-temperature stress on gene expression related to photosynthesis in two jujube (*Ziziphus jujuba* Mill.) varieties

**DOI:** 10.1080/15592324.2024.2357367

**Published:** 2024-05-22

**Authors:** Lei Yang, Xiaojuan Yang, Bingqi Shen, Juan Jin, Lili Li, Dingyu Fan, Subina Xiaokelaiti, Qing Hao, Jianxin Niu

**Affiliations:** a Department of Horticulture, College of Agriculture, Shihezi University, Shihezi, Xinjiang, China; b Xinjiang Production and Construction Corps Key Laboratory of Special Fruits and Vegetables Cultivation Physiology and Germplasm Resources Utilization, Shihezi, Xinjiang, China; c Institute of Horticulture Crops, Xinjiang Academy of Agricultural Sciences, Urumqi, Xinjiang, China; d The State Key Laboratory of Genetic Improvement and Germplasm Innovation of Crop Resistance in Arid Desert Regions (Preparation), Key Laboratory of Genome Research and Genetic Improvement of Xinjiang Characteristic Fruits and Vegetables, Scientific Observing and Experimental Station of Pomology (Xinjiang), Urumqi, Xinjiang, China

**Keywords:** High-temperature, jujube, photosynthesis, microstructure, photosynthetic gene

## Abstract

Elevated temperatures critically impact crop growth, development, and yield, with photosynthesis being the most temperature-sensitive physiological process in plants. This study focused on assessing the photosynthetic response and genetic adaptation of two different heat-resistant jujube varieties ‘Junzao’ (J) and ‘Fucuimi’ (F), to high-temperature stress (42°C Day/30°C Night). Comparative analyses of leaf photosynthetic indices, microstructural changes, and transcriptome sequencing were conducted. Results indicated superior high-temperature adaptability in F, evidenced by alterations in leaf stomatal behavior – particularly in J, where defense cells exhibited significant water loss, shrinkage, and reduced stomatal opening, alongside a marked increase in stomatal density. Through transcriptome sequencing 13,884 differentially expressed genes (DEGs) were identified, significantly enriched in pathways related to plant-pathogen interactions, amino acid biosynthesis, starch and sucrose metabolism, and carbohydrate metabolism. Key findings include the identification of photosynthetic pathway related DEGs and HSFA1s as central regulators of thermal morphogenesis and heat stress response. Revealing their upregulation in F and downregulation in J. The results indicate that these genes play a crucial role in improving heat tolerance in F. This study unveils critical photosynthetic genes involved in heat stress, providing a theoretical foundation for comprehending the molecular mechanisms underlying jujube heat tolerance.

## 1. Introduction

Rising global temperatures have intensified heat stress, a major limiting factor in global crop cultivation and yield.^1^ Plants have evolved multiple strategies to combat high temperatures at the cellular, physiological, and molecular levels to effectively reduce the negative effects of high-temperature stress on crop growth and development.^2,3^ Photosynthesis is one of the most sensitive physiological processes in plants to temperature change.^4^ The increasing adaptability of plants to high-temperature stress is related to the production of various hormones, and antioxidants, as well as dynamic membrane regulation and stomatal closure.^5,6^ Heat stress reduces leaf stomatal conductance, thus controlling the transpiration rate and photosynthetic rate.^7^ When the temperature is within the range required for plant growth, warming can increase stomatal conductance and intercellular CO_2_ concentration, thereby promoting photosynthesis. However, when the temperature exceeds the range required for plant growth, it leads to a decrease in net photosynthetic rate and water use efficiency.^8^

High-temperature stress not only harms the external morphology of the plant body but also affects the microstructure of plant cells. Under continuous high-temperature stress, mass wall separation will appear in most parts of plant cells and even lead to the rupture and disintegration of the vacuole membrane; the chloroplast will be destroyed; The outer mitochondrial membrane ruptures and gradually disintegrates, and its function is disrupted.^9^ Heat stress, at the molecular level, mainly changes the way genes participate in signaling pathways but also changes the transcriptional regulation and expression of genes in various pathways at the molecular level.^10^ Previous studies have found that gene expression levels induced by heat stress are regulated by transcription factors (TFs).^11^ High-temperature stress inhibits the photosynthetic process by reducing the enzyme activity of chloroplast protein complexes.^12^ For example, ribulose 1,5-diphosphate carboxylase and Rubisco-activase are high-temperature-sensitive enzymes.^13,14^ Disruption of high-temperature stress-mediated photosynthesis leads to the accumulation of reactive oxygen species (ROS), which leads to the oxidation of proteins, lipids, carbohydrates, and DNA, ultimately acting together to kill plant cells.^15,16^

Jujube (*Ziziphus jujuba* Mill.) belongs to the family Rhamnaceae and is a traditional tree native to China.^17^ The planting area of China is approximately 3.25 million hectares.^18^ It is widely cultivated in many arid and semi-arid marginal lands in China because of its superior drought resistance, salt and alkali resistance and barren resistance, and is an important source of income for nearly 20 million farmers in China.^19^ In recent years, high temperature stress has obviously affected the growth of jujube and has attracted people’s attention. Some differentially expressed genes responsive to high temperature stress were identified.^20^ The expression of *ZjALDH*^21^ and *ZjBAM*^22^ gene families in response to high temperature stress was analyzed. The elevated temperature (1.5–2.5°C than normal temperature) can increased the fruit sugar content, sugar-acid ratio, anthocyanins, flavonoids and carotenoids content.^23^ However, little is known about the gene expression related to photosynthesis in jujube under high temperature stress. In this study, two different heat-resistant jujube varieties^24^ that we reported were used as experimental materials to comparing their phenotypes, photosynthetic physiology, and transcriptional changes under high temperature stress. The aim was to analyze and study the expression changes of relevant differential genes in the photosynthetic pathway in response to high-temperature stress in jujube, and to tap into potential heat-stressed photosynthesis-related genes and transcription factors. This study contributes to a better understanding of the defense mechanisms associated with heat tolerance in jujube.

## 2. Materials and methods

### 2.1. Experimental materials

The test materials were 2-year-old J(Junzao, a heat sensitive variety) and F(Fucuimi, a heat-resistant variety) jujube-grafted potted seedlings, and the planting containers were 10 L plastic flowerpots with good management. The tree height was 80 cm, the crown width was 50 ~ 75 cm, and the trunk circumference was 6 cm. During heat stress, 400 ml of water per pot was provided at 20:00 per day, and 200 ml of Hoagland nutrient solution was provided every other day. Potted seedlings were placed in the culture room installed with an intelligent temperature control heater, the temperature was controlled within 42 ± 2°C, high temperature (42℃) stress treatment was carried out for 13 hours from 8 to 21 o ‘clock every day, The remaining 11 hours were spent on night culture at a temperature of 30°C. The photosynthetically effective radiation (PAR) was set to the average jujube light saturation point of 1520 μmol m^−2^ s^−1^. Real-time monitoring during the test using LUGER L95–6 temperature and humidity logger, Measurement of air temperature (Ta°C), relative humidity of air (RH%) in the greenhouse at 10 min intervals.

### 2.2. Sample Collection

Random J and F plants were selected to determine jujube growth potential. For each tree, including the control (0 d), sampling took place at 1 d, 3 d, 5 d, and 7 d after high-temperature stress treatment, and the sampling time was 14:00 each day. Leaves from the central lateral secondary branches (2 ~ 5) with mature function were collected and wiped with dust paper to clean the leaf surface of impurities. Sections with diameters of 6 mm were taken from both sides of the main vein leaves and placed into FAA solution (70% ethanol) in a 4℃refrigerator for preservation for electron scanning observation.

Furthermore, two leaves of the same position at each time point (*n* = 3) were collected, and the surface impurities of the leaves were washed with precooled distilled water. Then, the samples were cut into small pieces on dust-free paper, loaded into a liquid nitrogen precooled chilled storage tube, frozen in liquid nitrogen for 5 min, and stored in a −80°C refrigerator for transcriptome sequencing.

### 2.3. Observation of photosynthetic index and microstructure

The leaf photosynthetic indexes of 0 d, 1 d, 3 d, 5 d, and 7 d were determined by a Li100 6400XT portable gas exchange analyzer at the time point of high-temperature stress (*n* = 5). The change in chlorophyll content under different treatments was determined by a hand-held chlorophyll analyzer.

After dehydration, the blades being used to observe microstructure were freeze-dried to the critical point with an alcohol dilution series and then glued to the loading platform with conductive tape. Samples were coated with a Pt film using an ion sputtering instrument (Hitachi E-1045), and a SUPRA 55VP scanning electron microscope (Zeiss, German) was used for observation of leaves at an accelerating voltage of 2.00 Kv.

### 2.4. Transcriptome sequencing and data analysis

Leaves of two varieties from each treatment time (*n* = 3) were taken and total RNA was extracted using the RNA prep Pure polysaccharide polyphenol plant total RNA extraction kit. The purity of the extracted RNA was assessed by a Nano Drop 1000 spectrophotometer (Thermo Fisher Scientific, Wilmington, DE, USA). RNA concentration was determined by using a Qubit® 2.0 Flurometer (Life Technologies, Carlsbad, CA, USA). RNA integrity was assayed using the RNA Nano 6000 Assay Kit for the Agilent Bioanalyzer 2100 system (Agilent Technologies, Santa Clara, CA, USA). Then cDNA libraries were constructed, and after the library check was qualified, different libraries were pooled according to the target downstream data volume, and 30 cDNA libraries were sequenced using Illumina. High-quality clean reads were filtered from the raw reads and then sequence aligned with the reference genome: GCF_000826755.1. Sequence alignment maps were generated by TopHat2 and transcripts were assembled by Cufflinks (http://cufflinks.cbcb.umd.edu/).^25^ DEGs from different libraries were analyzed using the FPKM (fragments per kilobase transcribed per million mapped reads) method and screened for DEGs using edgeR_DESeq2 software.^26^ DEGs were identified based on differential fold change (FC ≥ 2) and false discovery rate (FDR ≤0.01). We also performed KEGG significance enrichment analysis of differential genes (*p* < 0.05) to obtain the functional clustering and metabolic pathways of differential genes in J and F jujube under high-temperature stress and demonstrated the differences in expression patterns of J and F jujube through heatmaps, and performed WGCNA analysis of the sequencing data.

### 2.5. Quantitative real-time PCR (qRT-PCR) validation

To verify the reliability of the transcriptome sequencing data, qRT‒PCR was used to verify the screened DEGs, referring to the Bu method.^27^ Gene sequences were obtained from NCBI (http://www.ncbi.nlm.nih.gov/genome/), Primer 3 software (http://frodo.wi.mit.edu/) was used to design primers, *ZjActin* was used as an internal primer,^28^ and all sequence primers are shown in Table S1. The same RNA samples used for transcriptome sequencing were used for qRT‒PCR validation. Fluorescence quantitative PCR was performed according to the SYBR® Green PCR Master Mix kit (Applied Biosystems, GA, USA), and the reaction system was 20 μl with the following PCR procedure: initial incubation at 95°C for 15 min, denaturing at 95°C for 10 s, annealing at 55°C for 20 s, and extension at 72°C for 30 s; 40 cycles were performed. The relative gene expression was calculated by the 2^−ΔΔCt^ method.^29^

### 2.6. Statistical analysis

SPSS 20.0 statistical software (SPSS, IL, USA) was used for one-way analysis of variance (ANOVA). Duncan’s multiple test was used to analyze the differences between the mean values, and the results were considered significant when the *p*-value ≤0.05. Data plotting was performed using GraphPad Prism 8.

## 3. Results

### 3.1. Environmental changes within the chamber before and after high-temperature treatment

**Figure 1.**
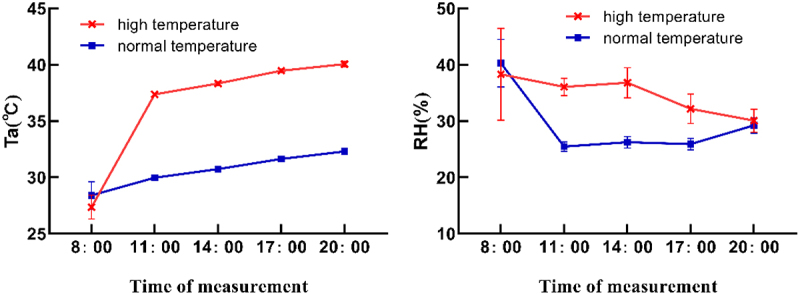
Diurnal change of temperature and humidity before and after high temperature treatment.

Light intensity in the greenhouse before and after the high-temperature treatment was 500 μmol m^−2^ s^−1^. The daily variation of temperature and humidity in the culture room before and after high temperature treatment and the growth environment of date seedlings during the test period are shown in Figure 1. The daily variation curve of inter-incubation temperature Ta (°C) showed a gradual increasing trend, Maximum temperature of 32.5°C before treatment and 41.1°C after treatment. Both reach their maximum at 20:00. The daily change curves of air relative humidity RH/% in the incubation room before and after treatment were completely different, The relative humidity of the air was maximum at 33.93% at 8:00 a.m. before the treatment and then showed a sharp downward trend. The minimum value of 24.88% was reached at 11:00, and the relative humidity of the air kept increasing with the prolongation of light time, The daily trend of air relative humidity in the observed time period after heating was: decreasing, then increasing, and then gradually decreasing, reaching a peak of 36.8% at 14:00, and decreasing to a minimum value of 29.3% at 20:00.

### 3.2. Phenotypic and photosynthetic responses of two varieties to continuous high-temperature stress

**Figure 2.**
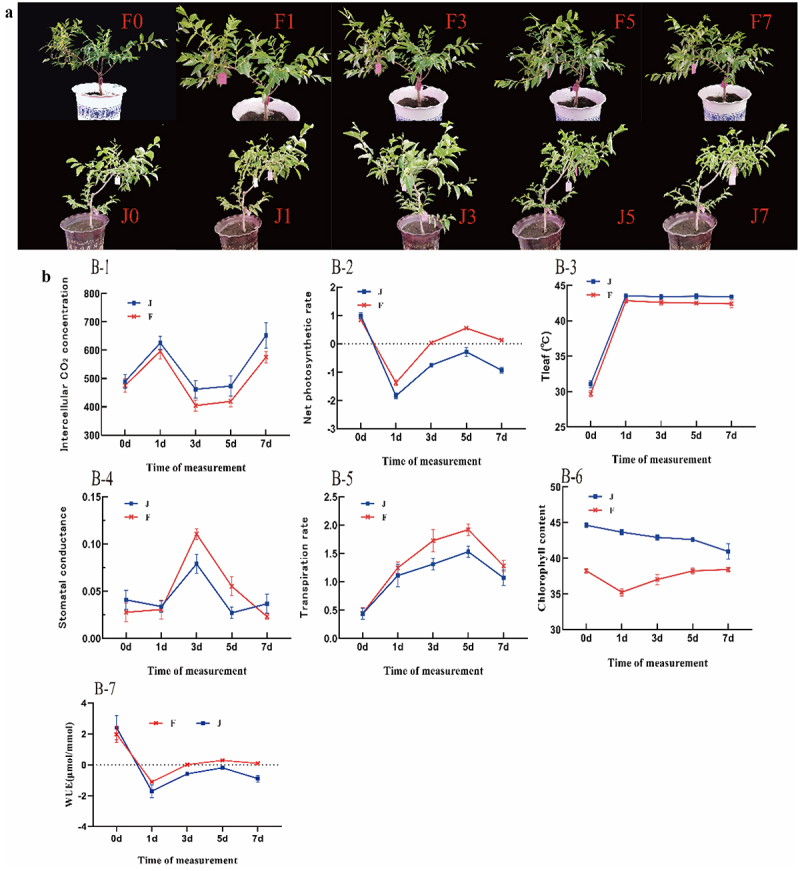
Photosynthetic response to high-temperature stress: (a) Phenotypes under dynamic high-temperature stress in F and J. (b) Photosynthetic parameters under high-temperature stress in F and J. The error bars represent standard error.

**Figure 3.**
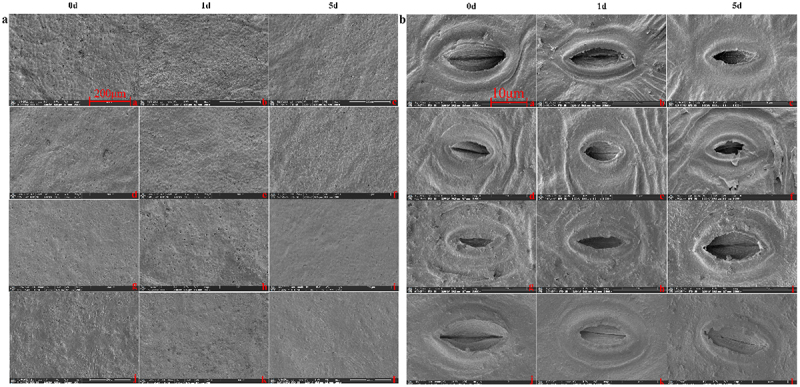
SEM results of two varieties leaves under high-temperature stress.

Note: Fig. a, b, c and Fig. g, h, i show the 0, 1, 5 days of F and J at room temperature, respectively. Fig. d, e, f and Fig. j, k, l show the 0, 1, 5 days of F and J under high-temperature stress.

**Figure 4.**
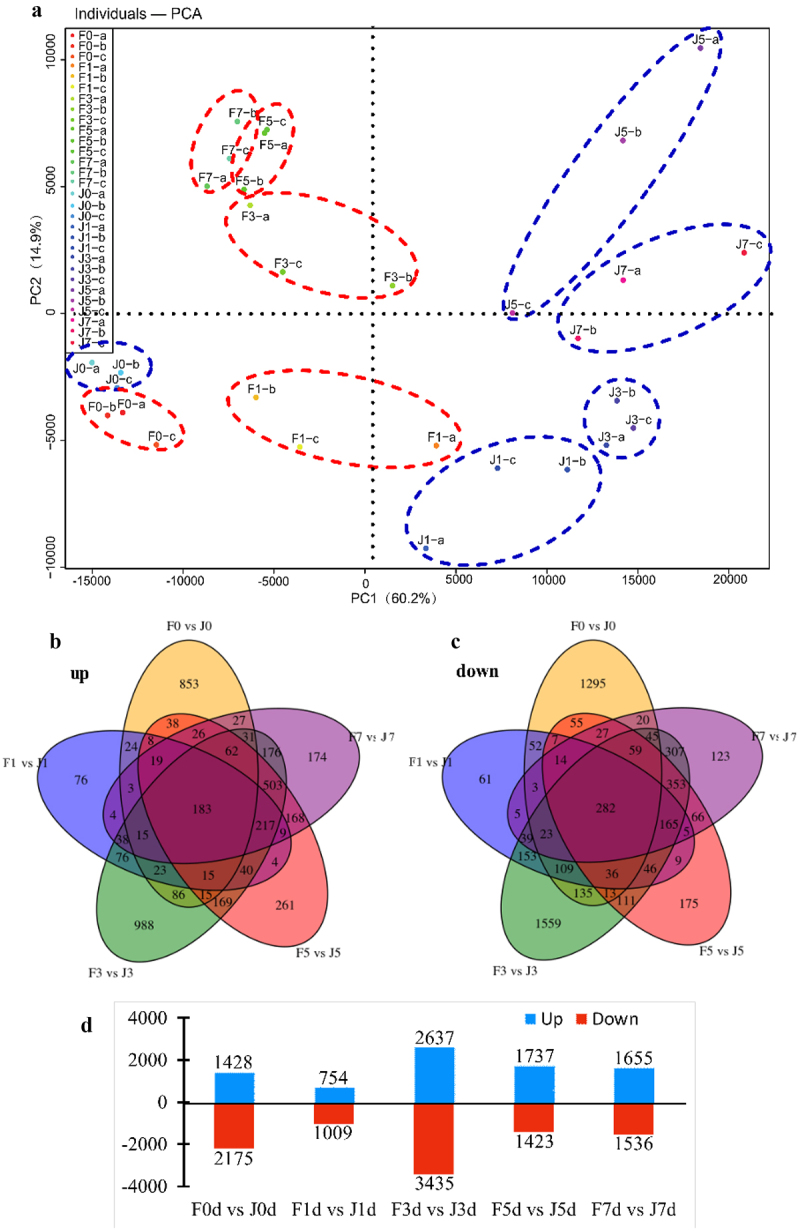
Comparison of transcriptome data of two varieties under different high temperature treatments. (a) PCA analysis. (b,c) Venn diagram of DEGs in different comparison groups. (d) Statistical bar chart of the number of DEGs by comparing F0 vs J0, F1 vs J1, F3 vs J3, F5 vs J5, and F7 vs J7. Blue represents the number of upregulated DEGs, while red stands for downregulated DEGs.

**Figure 5.**
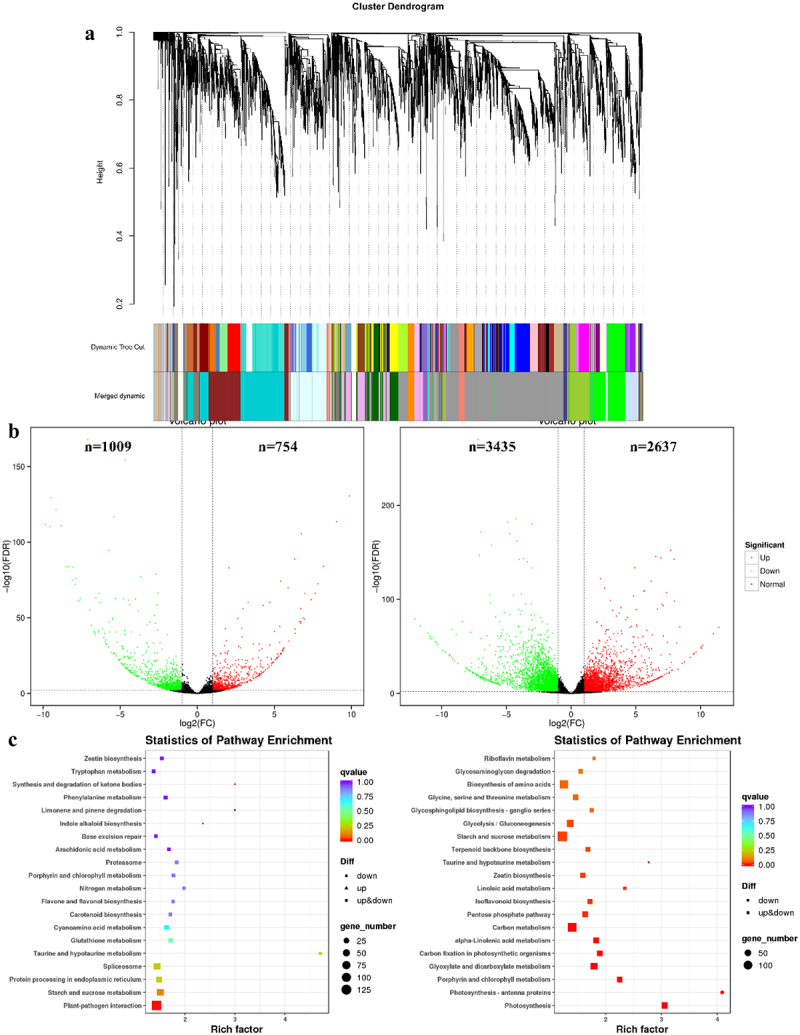
Comparative transcriptomic analysis. (a) Clustering analysis of WGCNA co-expressed genes. (b) Comparison of F1 vs J1 and F3 vs J3 volcano plots, with green dots representing down-regulated genes and red dots representing up-regulated genes. (c) Comparison of F1 vs J1 and F3 vs J3 KEGG-enriched top 20 pathways.

**Figure 6.**
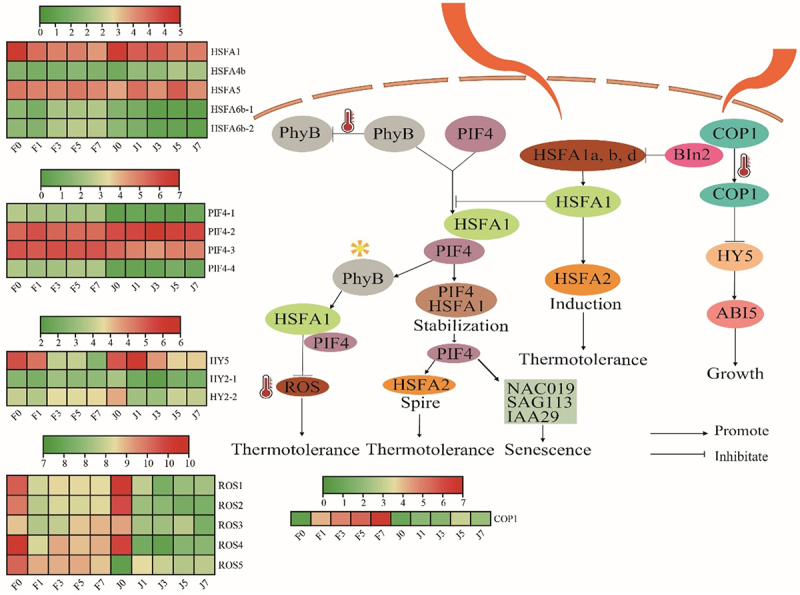
Expression analysis of the photosynthesis pathway and its core genes and transcription factors. HSFA, heat shock transcription factor; PIF4, phytochrome interacting factor 4; ROS, reactive oxygen species; COP1, constitutive photomorphogenic1; HY, elongated hypocotyl; phyB, phytochrome B; BIN2, brassinosteroid insensitive 2; ABI5, abscisic acid-insensitive 5; NAC019, N-Acetyl cysteine019; SAG113, surface antigen 113; IAA29, indole acetic acid 29. The colors of squares indicate the fold changes of the DEGs (red is upregulated and green is downregulated).

**Figure 7.**
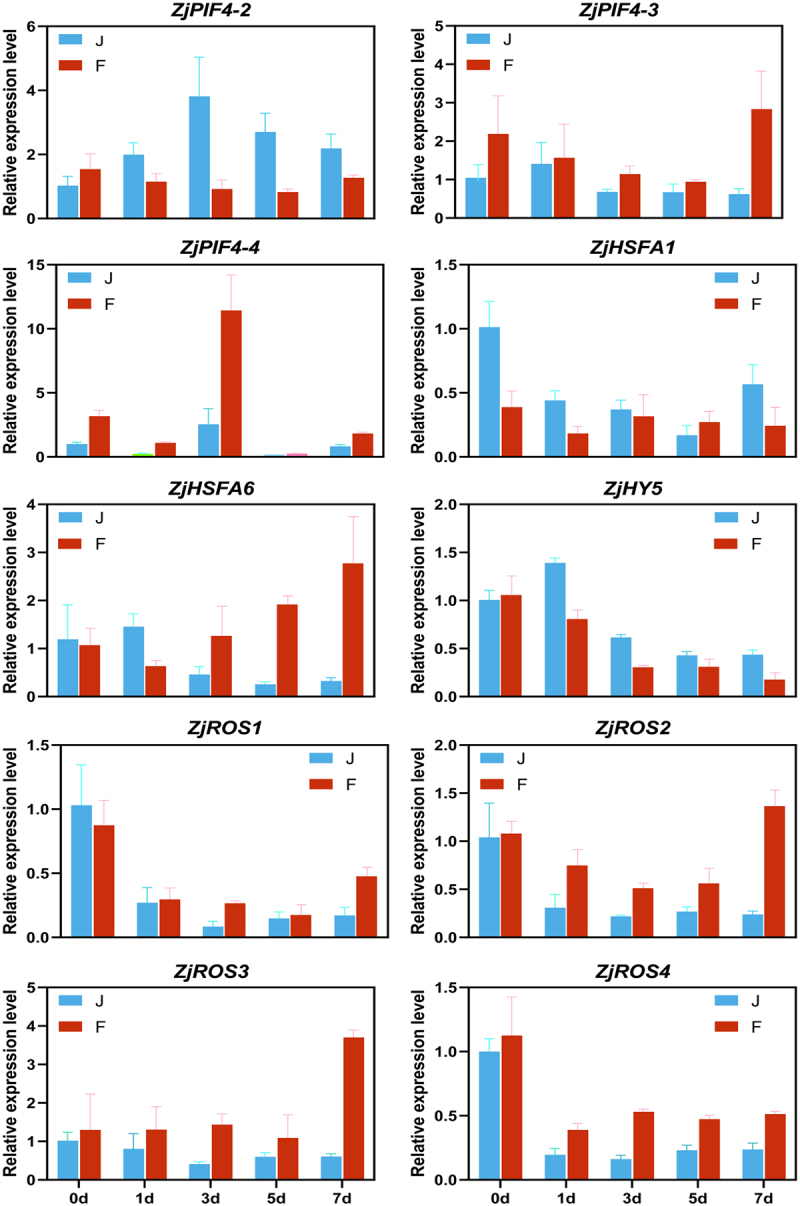
Results of qRT-PCR validation. Each column represents the average of three biological replicates, with standard errors indicated by vertical bars.

According to the genetic characteristics of the plants, different leaves of the same tree were different in structure, thickness, and chlorophyll content.^30^ Figure 2a shows that F exhibited no significant phenotypic changes under high temperature for 7 days. In contrast, J showed leaf wilting and curling with prolonged heat exposure, leading to complete leaf wilting, curling, and shedding after 7 days. As shown in Figure 2b, during high-temperature stress, the intercellular CO_2_ concentration of the leaves of F was always lower than that of J (Figures 2b–1), and the stomatal conductivity of F was greater than that of J (Figures 2B–4); therefore, the stomata of F leaves may not completely close and still undergo gas exchange, indicating CO_2_ utilization is high, water utilization is also relatively high(Figures 2b–7), and the photosynthetic function is satisfactory, making F photosynthesis stronger than J photosynthesis(Figures 2b–2). In the absence of high-temperature stress, the leaf transpiration rates of two varieties were equal and minimal, With the extension of heat stress time F was always higher than J. This may be due to transpiration lowering the temperature of the tree, so that F receives less heat damage(Figures 2b–5), The higher transpiration rate carries away some of the heat, so during high-temperature stress F leaf temperature is always lower than J(Figures 2b–3), that the chlorophyll content of F decreased sharply to the lowest level when the heat stress lasted for 1 d and then increased continuously with the increase in the number of days of heat stress, while the chlorophyll content of J always decreased with the increase in the number of days of heat stress(Figures 2b–6).

### 3.3. Effects of high temperature stress on microstructure of leaves

Utilizing 200× and 3500× magnification, the study captured the stomatal characteristics of these varieties over 0, 1, and 5 days of high-temperature exposure. These detailed observations are comprehensively presented in Figure 3. The analysis reveals a variety-specific response to thermal stress. J exhibited more pronounced changes than its counterpart, characterized by significant stomatal closure and guard cell deformation, as evidenced by the diminishing stomatal opening rate.

F maintained a stable stomatal density, akin to the unstressed control group, while J showed an increase, particularly after one day of stress exposure (Table 1). Additionally, the stomatal opening rates in both declined as the stress duration increased, with F showing a slight recovery at the five days mark, unlike the steady decline observed in J (Figure 3). These findings not only distinguish the stress responses between F and J, but also provide crucial insights into their stomatal adaptation strategies under prolonged heat stress.

**Table 1.** Effects of high-temperature stress on the stomatal properties of jujube leaves.

Varieties	Temperature	Time after treatment	Stomatal open rate（%）	Stomatal open number（a）	Total number of stomata（a）	Stomatal density (a/mm^2^)
Fucuimi	Normal temperature	0d	81.13±0.91d	68.67±0.33c	84.67±1.20a	313.73±4.45a
		1d	80.61±2.59d	69.00±1.52c	85.67±1.45a	317.43±5.38a
		5d	79.81±2.51d	68.33±2.02c	85.67±1.76a	317.43±6.53a
	High temperature	0d	78.82±1.58d	68.00±1.15c	86.33±2.02a	319.90±7.51a
		1d	71.39±4.51c	61.00±2.64ab	85.67±1.76a	317.43±6.53a
		5d	75.30±0.83cd	66.00±0.58bc	87.67±0.88a	324.84±3.27a
Junzao	Normal temperature	0d	69.75±3.18c	76.67±3.84d	110.00±3.60bc	407.59±13.36bc
		1d	71.18±0.77c	77.33±1.33de	108.67±2.02b	402.66±7.51b
		5d	73.46±3.55cd	80.33±2.33e	103.67±2.40bc	401.18±8.90bc
	High temperature	0d	70.77±0.57c	80.00±3.05de	113.00±3.51bc	418.71±13.01bc
		1d	58.17±1.71b	67.67±2.18c	116.33±1.66c	431.06±6.17c
		5d	50.19±1.39a	56.33±0.88a	112.33±2.02bc	416.24±7.51bc

### 3.4. Transcriptomic analysis of two varieties under high-temperature stress

To unravel the transcriptomic responses of jujube varieties to high-temperature stress, an extensive RNA-Seq analysis was conducted over periods of 0, 1, 3, 5, and 7 days. This rigorous study included three biological replicates at each interval, culminating in the creation of 30 RNA-Seq cDNA libraries. Principal component analysis (PCA) validated the reproducibility among replicates and highlighted distinct transcriptional changes under varying degrees of heat stress, as illustrated in Figure 4a.

The Number of DEGs in different sample comparisons show in Table S2. The study primarily focused on identifying DEGs between the J and F varieties under equivalent stress conditions (Figure 4). Findings revealed that in the initial comparison (F0d vs. J0d), there were 1,428 genes upregulated and 2,175 downregulated. As stress duration increased, the number of regulated genes fluctuated, with 754 upregulated and 1009 downregulated genes noted in F1d compared to J1d. The trend continued with significant gene expression variations observed in subsequent comparisons (F3d vs. J3d, F5d vs. J5d, and F7d vs. J7d). The Venn diagram analysis pinpointed 183 DEGs consistently upregulated and 282 consistently downregulated across all treatments, suggesting a core stress response mechanism. The differential expression patterns underscore the varietal distinction in temperature tolerance, particularly highlighting F resilience.

Furthermore, a unique set of 174 upregulated and 123 downregulated genes emerged after 7 days of stress, displaying no overlap with earlier stress responses. This specificity indicates complex gene regulation dynamics at play, contributing to the distinct adaptive strategies of F and J under prolonged heat exposure.

### 3.5. Comparative analysis of DEGs between two varieties

WGCNA analysis categorized 13,884 DEGs into 21 major modules (Figure 5a). A total of 224.58Gb Clean Data was obtained from transcriptome sequencing, and the Clean Data of each sample reached 5.74Gb (Table S3). Physiological metrics-based assays analyzed transcriptome data only for 1 d and 3 d of high temperature. The number of DEGs identified in the different groups was 1763 and 6072 by comparative analysis of F1 vs. J1 and F3 vs. J3, respectively. The volcano plot shows the clustering of up-and down-regulated DEGs, with 1009 down-regulated genes and 754 up-regulated genes in J1 compared to F1, and 3435 down-regulated genes and 2637 up-regulated genes in J3 compared to F3, respectively (Figure 5b). KEGG pathways were enriched for DEGs under high-temperature stress for these two days, and the 20 pathways with the highest degree of enrichment were extracted. The results showed significant enrichment in the pathways plant-pathogen interactions, amino acid biosynthesis, starch and sucrose metabolism and carbon metabolism (Figure 5c), It was shown that these DEGs were closely related to the changes in various indicators of photosynthesis under high-temperature stress. Among them, starch and sucrose metabolism and carbon metabolism are closely related to photosynthesis. F0 vs J0, F5 vs J5, F7 vs J7, KEGG enrichment analysis and gene expression distribution maps in Fig. S1-S6.

### 3.6. Identification of key genes in the photosynthetic pathway

The efficiency of photosynthesis is critical to plant adaptation. More efficient photosynthesis leads to better plant adaptation.^31^ The results in Figure 6 shows that high-temperature conditions during the daytime lead to a shift of *phyB* to an inactive form, while on the one hand high temperature induces a large accumulation of*HSFA1s* proteins in the light, On the other hand, high-temperature-induced *COP1* entry into the nucleus to inhibit the binding of *BIN2* to *HSFA1s*, thereby promoting the entry of *HSFA1s* into the nucleus. However, extremely high temperatures at 42°C inhibit the activity of *COP1*, which in turn stabilizes *HY5* and directly induces the expression of *ABI5* to inhibit growth and development, The heatmap showed that the expression of *COP1* was significantly higher in F than in J with the high-temperature stress increased. In the nucleus, *HSFA1s* prevent *PIF4* degradation by photoactivated *phyB* by forming a stabilizing protein complex with *PIF4*. This promotes the thermal morphogenesis process in the light, and the light-activated transcription factor PIF4 directly induces the expression of *HSFA2*, which makes the new leaves thermotolerant. In contrast, PIF4 also triggers the expression of senescence-related genes (*NAC019*, *SAG113*, and *IAA29*) in old leaves, thereby inducing leaf senescence. In addition, *HSFA1s* can also directly induce *HSFA2* expression under high daytime temperatures, resulting in plants acquiring strong high-temperature stress tolerance. Meanwhile, light-activated *phyB* induces the expression of the reactive oxygen species scavenging enzyme gene *APX2* by regulating the transcription factor HSFA1, which mediates the de-oxidation of *ROS* and thus enhances the heat tolerance of plants. In summary, HSFA1s, as the core regulatory elements, control the processes of thermal morphogenesis and response to heat stress.

### 3.7. qRT-PCR validation

To confirm the validity of the RNA-seq data, 10 genes with significant differences in the photosynthetic pathway were selected for qRT-PCR validation. The results showed consistent expression patterns between qRT-PCR and RNA-seq data (Figure 7).

## 4. Discussion

The photosynthesis indexes showed that the influence of high-temperature stress on the heat-sensitive variety J was more obvious than that on the heat-resistant variety F. During high-temperature stress, the intercellular CO_2_ concentration of F leaves was always lower than that of J, and the stomatal conductivity of F was higher than that of J. It was speculated that the leaves of F were still undergoing gas exchange because their stomata were not completely closed, timely replenishment of CO_2_ consumed by photosynthesis of F, so the CO_2_ utilization rate was higher. In addition, the water utilization rate was relatively high, meeting the requirements of photosynthesis and making the photosynthesis of F stronger than that of J, which is consistent with the research results of Pakdel et al..^32^ When there was no high-temperature stress, the transpiration rate of two varieties was the same and the lowest. With the increase of high-temperature stress time, the change curve of the two varieties showed a bi-peak shape, and F was always higher than J, while leaf temperature was lower than that of J, suggesting that higher transpiration rates helped to reduce leaf temperature in F, thereby maintaining the physiological activity of its leaf cells and enabling it to maintain normal growth, The opposite is true for J, a speculation that is corroborated by the phenotype (Figure 2a). By studying the differences in photosynthetic parameters between J and F, the influence of photosynthesis on the two jujube varieties under the same cultivation and management conditions are clearer. Chlorophyll is an important pigment related to photosynthesis and exists in all photosynthetic organisms.^33^ The results of this experiment showed that the chlorophyll content of F decreased sharply to the lowest level at 1 d of high-temperature stress and then increased with the increase in the number of high-temperature stress days, while the chlorophyll content of J always decreased with the increase in high-temperature stress days, indicating that the effect of high-temperature stress on the chlorophyll content of J was significantly higher than that of F. Chlorophyll absorbs energy from light and is then used to convert carbon dioxide into carbohydrates for plant growth, so it may be that the reduction in chlorophyll content reduces the photosynthetic rate of J, while F is almost unaffected.

Stomata are the main channels for water and CO_2_ exchange between plants and the outside world.^34^ By regulating the amount of gas through the opening and closing of guard cells, pore size directly affects crop photosynthesis and transpiration,^35^ which has an extremely important physiological role. The changes in stomata are closely related to changes in the external environment.^36^ In this study, with the increase of heat stress time, the stomatal characteristics of both varieties were affected, and the effect on J was more significant, which was mainly manifested as shrinking and drying of guard cells and a significant decrease in stomatal opening rate. The results indicated that J leaves suffered great damage under high-temperature stress, and the normal physiological effects were greatly inhibited. This is consistent with the results of rice leaves.^37^ When exposed to high-temperature stress, plants generally optimize gas exchange efficiency by changing the stomatal size, density, openness and opening rate of leaves.^38^ However, some researchers believe that high temperatures will not cause changes in stomatal density and other characteristics of plant leaves.^39^ In this study, with the extension of high-temperature stress time, compared with jujube trees growing in parallel without stress treatment, the stomatal density of F showed no significant change, while that of J increased significantly, especially after 1 d of high-temperature stress. This may be because the stomatal opening rate of the heat-resistant variety F did not change significantly under high-temperature stress and therefore did not affect the normal gas exchange, while the stomatal opening rate of J continued to decline with the increase in the number of days of high-temperature stress. So the transpiration rate F is always higher than J. Higher transpiration reduces the temperature of the tree, so the leaf temperature of F is always lower than J during high temperature stress (Figure 2-B-3), and therefore F suffers less heat damage and is more tolerant to high temperatures.

To further explore the mechanism of heat tolerance stress in jujube trees at the molecular level, transcriptome sequencing analysis was carried out in this study, and the differences in gene expression between the heat-resistant variety F and the heat-sensitive variety J were revealed. A good correlation of the three biological replicates for each sample was verified by PCA analysis (Figure 4), This supports the reproducibility of the transcriptome data and the significant difference in transcript levels between the two varieties in response to high-temperature stress. Based on the observation of phenotypic and physiological indexes of the two varieties under high-temperature stress, the comparative analysis of transcriptome data at 1 d and 3 d showed that the number of differential genes increased significantly at heat stress 3 d. Previous studies have found that there are significant differences in the expression of heat- resistant and heat-sensitive laver varieties under high-temperature stress.^40,41^ The results of KEGG enrichment analysis showed significant enrichment in the pathways of “plant-pathogen interactions”, “amino acid biosynthesis”, “starch and sucrose metabolism” and “carbon metabolism” under high-temperature stress. This is similar to the results of our identification of photosynthetic complex genes in two other jujube varieties with different heat resistance.^20^ Therefore, it is hypothesized that although J initiated individual genes compatible with high temperature under sustained high-temperature stress, but it does not or rarely synthesizes new proteins during stress, thus lacking the ability to withstand high temperature stress.

The transcription factor HSFA, as a central regulatory element in the photosynthetic pathway in response to high-temperature stress, controls both thermal morphogenesis and heat stress response. High-temperature stress inhibits the photosynthetic process by reducing the activity of chloroplast protein complex enzymes.^12^ The main reason is that high temperature inhibits photosynthesis and leads to the accumulation of ROS, which leads to the oxidation of proteins, lipids, carbohydrates, and DNA and ultimately to the death of plant cells,^15,16^ This is consistent with the results of this study. These results may help to explain why F is more tolerant to high-temperature stress than J and aid in the exploration of its heat resistance mechanism. In addition, PsaK and PsaG are also important core proteins in PSI and play an important role in the phototrapping complex of PSI.^42,43^ Under salt and alkali stress, six proteins related to photosynthesis, such as PsaK, were significantly upregulated in malonia, and these proteins could act as regulators of the PSI repair system.^44^ A total of 10 DEGs and transcription factors in photosynthesis in response to high-temperature stress were identified in this study, including three PIF4 transcription factors, two HSFA transcription factors, four *ROS* genes, and one *HY5* gene, and their expression was upregulated in F after high-temperature stress, However, downregulated expression of these genes was also observed in J (Figure 6). It was suggested that high temperature stress induced the expression of these genes, which helped to stabilize the structure of PSII and PSI from being damaged by high temperature. Therefore, it is hypothesized that up-regulated photosynthesis-related genes play an important role in heat tolerance of jujube.

## 5. Conclusions

In summary, our study investigated the responses of two jujube varieties J and F to continuous high-temperature stress, revealing notable distinctions in phenotypic, photosynthetic, and molecular aspects. F exhibited enhanced heat tolerance, maintaining stable phenotypic characteristics, higher photosynthetic efficiency, and unique transcriptomic responses. Chlorophyll content dynamics and detailed leaf microstructure observations further emphasized the differential adaptive strategies of two varieties. Transcriptomic analysis identified key genes, with WGCNA highlighting modular gene expression patterns. Notably, F demonstrated higher expression of COP1 in the photosynthetic pathway, indicative of its potential contribution to improved heat tolerance. qRT-PCR validation confirmed the reliability of our findings. Overall, these insights deepen our understanding of the mechanisms governing heat tolerance in jujube, laying the foundation for future exploration of genetic and physiological factors influencing plant responses to environmental stress.

## Supplementary Material

Supplementary_tables.docx

## Data Availability

The raw sequence data reported in this paper have been deposited in the Genome Sequence Archive (Genomics, Proteomics & Bioinformatics 2021) in National Genomics Data Center (Nucleic Acids Res 2022), China National Center for Bioinformation/Beijing Institute of Genomics, Chinese Academy of Sciences (BioProject ID: PRJCA016517) that are publicly accessible at https://ngdc.cncb.ac.cn/.
