# Supplementary material for: Effects of high-temperature stress on gene expression related to photosynthesis in two jujube (Ziziphus jujuba Mill.) varieties
Source: Plant Signal Behav. 2024 May 22;19(1):2357367. doi: 10.1080/15592324.2024.2357367 (PMC11139005; doi:10.1080/15592324.2024.2357367)
Supplement: Supplementary_tables.docx [file KPSB_A_2357367_SM1234.docx]

**Table S1.** Genes and primer pairs for real-time quantitative PCR

| **Genes** | **Sequence (5'-3')** |
| --- | --- |
| PIF4-2 | Sense: TAGCAGGGTGTCCAGCAATG  Antisense: GCTCAGTCGAAGGCTCAGTT |
| PIF4-3 | Sense: GCTTGAATCCAGGCCTTCCT  Antisense: ATCTCCATAAGCTGCGACCG |
| PIF4-4 | Sense: ACAGCTTTGTCCCGTTCTCA  Antisense: GCTTCCAAGCCTTTTGCCTC |
| HY5 | Sense: TGCTCAACAAGCCCGAGAAA  Antisense: TGTTGAGGAGAACCTTGCGA |
| HSFA1 | Sense: GAACGAGGCAGCAAAAGCAA  Antisense: GCATCAGGATAGCAGACCCC |
| HSFA6 | Sense: GTTATTCGCAGCAGGGAGGT  Antisense: GGTTGGGTCATCCACCATGT |
| ROS1 | Sense: AGGAGAGTATGCTGGGCTGA  Antisense: AGGATTTGTCCCATGGGCTG |
| ROS2 | Sense: CACTCACCATCCCCACCTTC  Antisense: TTGTCGAGGGTCTCAACAGC |
| ROS3 | Sense: CGGTCCGTTTGGAGAAGTCA  Antisense: GCCGAACAGCTATAGCCACA |
| ROS4 | Sense: GCTGTTCACATTGCAGCCAA  Antisense: GAAGTAAGGGCTGGGGTTCC |

**Table S2.** Number of DEGs in different sample comparisons

| **DEGs** | **DEG Number** | **up-regulated** | **down-regulated** |
| --- | --- | --- | --- |
| F0d_vs_F1d | 797 | 318 | 479 |
| F0d_vs_F3d | 2656 | 1698 | 958 |
| F0d_vs_F5d | 5297 | 3100 | 2197 |
| F0d_vs_F7d | 4831 | 2988 | 1843 |
| F0d_vs_J0d | 3603 | 1428 | 2175 |
| F1d_vs_J1d | 1763 | 754 | 1009 |
| F3d_vs_J3d | 6072 | 2637 | 3435 |
| F5d_vs_J5d | 3160 | 1737 | 1423 |
| F7d_vs_J7d | 3191 | 1655 | 1536 |
| J0d_vs_J1d | 4139 | 2171 | 1968 |
| J0d_vs_J3d | 5846 | 3119 | 2727 |
| J0d_vs_J5d | 6330 | 3983 | 2347 |
| J0d_vs_J7d | 4801 | 2938 | 1863 |

**Table S3.** The data statistics of sample sequencing

| **Samples** | **Clean reads** | **Clean bases** | **GC Content** | **%≥Q30** |
| --- | --- | --- | --- | --- |
| F0-a | 28,628,379 | 8,549,788,050 | 44.53% | 94.65% |
| F0-b | 26,702,995 | 7,967,330,702 | 44.63% | 94.60% |
| F0-c | 19,245,224 | 5,737,977,904 | 44.52% | 93.97% |
| F1-a | 28,062,603 | 8,377,365,300 | 44.54% | 93.97% |
| F1-b | 27,426,035 | 8,183,268,874 | 44.64% | 94.40% |
| F1-c | 24,828,266 | 7,392,657,194 | 44.53% | 94.27% |
| F3-a | 19,375,993 | 5,776,961,458 | 44.68% | 94.75% |
| F3-b | 19,292,829 | 5,746,240,756 | 44.40% | 94.30% |
| F3-c | 22,819,028 | 6,808,465,838 | 44.51% | 94.34% |
| F5-a | 26,027,804 | 7,765,131,864 | 44.56% | 94.46% |
| F5-b | 27,031,420 | 8,061,708,746 | 44.53% | 94.15% |
| F5-c | 26,314,719 | 7,837,393,856 | 44.76% | 94.10% |
| F7-a | 37,573,367 | 11,201,847,118 | 44.67% | 94.27% |
| F7-b | 23,656,850 | 7,045,291,024 | 44.73% | 94.67% |
| F7-c | 26,390,908 | 7,863,047,430 | 44.72% | 94.82% |
| J0-a | 25,552,167 | 7,629,912,028 | 44.87% | 94.68% |
| J0-b | 19,404,796 | 5,778,788,236 | 44.73% | 94.23% |
| J0-c | 25,918,480 | 7,732,122,310 | 44.80% | 94.72% |
| J1-a | 26,798,323 | 7,995,547,152 | 44.36% | 94.69% |
| J1-b | 25,171,831 | 7,507,359,756 | 44.37% | 94.66% |
| J1-c | 26,630,710 | 7,929,908,970 | 44.49% | 94.43% |
| J3-a | 19,594,227 | 5,839,052,054 | 44.25% | 94.31% |
| J3-b | 26,032,902 | 7,770,328,060 | 44.14% | 94.49% |
| J3-c | 25,878,295 | 7,713,766,784 | 44.16% | 94.31% |
| J5-a | 27,967,837 | 8,335,536,204 | 44.24% | 94.21% |
| J5-b | 19,681,182 | 5,853,801,038 | 44.23% | 94.47% |
| J5-c | 27,102,555 | 8,079,772,042 | 44.27% | 94.58% |
| J7-a | 24,859,246 | 7,404,890,480 | 44.33% | 94.54% |
| J7-b | 21,675,038 | 6,440,268,428 | 44.28% | 94.56% |
| J7-c | 27,659,396 | 8,254,129,692 | 44.35% | 94.57% |

Note: (1) Samples: analysis numbers; (2) Clean reads: total number of paired-end reads in clean data; (3) Clean bases: total number of bases in clean data; (4) GC content: clean data GC content, namely, the percentage of G and C bases in the total bases of the clean data; (5) ≥Q30%: clean data are the percentage of bases with mass value greater than or equal to 30.
